# CB2 Receptor Agonist JWH133 Activates AMPK to Inhibit Growth of C6 Glioma Cells

**DOI:** 10.1515/biol-2019-0041

**Published:** 2019-07-22

**Authors:** Feng Wang, Jing Wang, Tong Zhao, Yi Zhang, Qian Li

**Affiliations:** 1 Hebei Collaborative Innovation Center for Cardio-cerebrovascular Disease, Shijiazhuang 050000, China; 2 Department of Physiology, Hebei Medical University, Shijiazhuang 050017, China; 3 Hebei Provincial Cancer Institute, Shijiazhuang 050011, China

**Keywords:** endocannabinoid receptor type 2, C6 glioma cells, JWH133, AMPK, cAMP-PKA, CaMKK

## Abstract

It has been reported that endocannabinoid receptor type 2 (CB2) agonist JWH133 inhibits the growth of C6 glioma cells, but the underlying mechanism has not yet been fully elucidated. We showed that JWH133 inhibited C6 cells growth, reduced cAMP production and inhibited PKA activity through CB2 receptor. Decrease of PKA activity stimulated CaMKKβ, and subsequently elevated phosphorylation of AMPKα at threonine 172 site. The activation of AMPKα induced changes of downstream proteins, including increase of P53 phosphorylation and P21 production, as well as decrease of mTOR phosphorylation, that eventually inhibited C6 cells growth.

## 1 Introduction

Cannabinoids are lipid mediators, originally isolated from the hemp plant Cannabis sativa, that play their effects by activating primarily two G-protein-coupled receptors: type 1 cannabinoid receptor (CB1) and type 2 cannabinoid receptor (CB2) [1]. The CB1 receptors are located in the brain and several peripheral tissues. On the other hand, the CB2 receptors are expressed primarily in the immune system, but their presence in the brain has also been demonstrated recently, for instance, in microglial cells, astrocytes and even some neuron subpopulations [2].

Glioma is the most common form of brain cancer, which results in the death of affected patients within months after diagnosis. Conventional therapies, including surgery, radiotherapy, chemotherapy and immunotherapy, are usually ineffective or just palliative [3]. Analyses of astrocytomas demonstrate that 70% of the tumors express CB1 and/or CB2 receptor, and CB2 receptor expression level correlates with tumor malignancy [4]. CB2 receptor is expressed at low levels in microglial cells under normal conditions, however, its overexpression is correlated with malignant tumor development [5]. In C6 glioma cells and astrocytoma xenografts, CB2 receptor agonist JWH-133 can reduce cell viability by 50% in vitro, while in vivo studies show a 71% decrease in tumor growth. This effect on tumor growth can be inhibited by the CB2 antagonist SR144528, but not the CB1-specific antagonist SR141716 [4].

However, mechanisms between CB2 receptor activation and glioma cells inhibition have not been fully elucidated. AMP-activated protein kinase (AMPK) is a metabolic-sensing protein kinase, which acts as an energy-sensor mainly in ATP-deprived conditions [6]. In the activated state, AMPK down-regulates several anabolic enzymes to shut down the ATP-consuming metabolic pathways. Many studies have shown that AMPK activation inhibits the proliferation of tumor cells, such as glioblastomas [7]. Furthermore, it has been reported that activation of CB2 receptor also leads to AMPK activation. Vara D et al. reported that CB2 receptor-selective agonists D9-THC and JWH-015 can reduce the viability of human hepatocellular carcinoma cell line HepG2 and HuH-7 via activating AMPK[8]. In addition, activation of CB2 receptor-mediated AMPK/CREB pathway is reported to reduce cerebral ischemic injury [9]. However, it remains unknown whether AMPK activation is involved in CB2 receptor-induced inhibition of glioma growth. Our current study indicated that CB2 receptor agonist JWH133 inhibited the growth of C6 glioma cells through activation of AMPK, and provided the underlying molecular mechanism.

## 2 Materials and methods

### 2.1 Chemicals and reagents

JWH-133 and ACEA was purchased from Tocris Bioscience (Westwoods Business Park Ellisville, Missouri, USA). The CB2 antagonist AM630 was purchased from Cayman Chemical (Ann Arbor, MI, USA). PKA inhibitor IV and 8-Bromo-cAMP were purchased from Santa Cruz Biotechnology (Dallas, Texas, USA.) Antibodies against CNR1, CNR2, phospho-AMPK alpha (Thr172), AMPK alpha, P21, phospho-p53 (Ser15), P53, phospho-mTOR (Ser2448) and mTOR were purchased from Affinity Biosciences (Cincinnati, OH, USA). Antibodies against p-LKB1, LKB1, PCNA and β-Actin were purchased from Abcam Trading Company Ltd (Pudong, Shanghai, China). Goat anti-rabbit IgG-HRP and goat anti-mouse IgG-HRP secondary antibodies were purchased from KPL (Gaithersburg, MD, USA). All the other chemicals were obtained from Sigma.

### 2.2 Cell culture

The C6 cells were maintained in DMEM (GIBCO, Shanghai, China), supplemented with a 10% FBS (GIBCO Shanghai, China), penicillin/streptomycin (1:100; BIOIND, Kibbutz Beit Haemek, Israel) in a 5% CO_2_ incubator at 37°C.

### 2.3 Cell viability assay

Cell viability was measured by the 3-(4,5-dimethylthylthiazol-2-yl)-2,5-diphenyltetrazolium bromide (MTT) method. Briefly, cells were collected and seeded in 96-well plates at a density of 5×10^5^ cells/cm^2^. Different seeding densities were optimized at the beginning of the experiments. 20 μl of MTT tetrazolium salt (Sigma, St. Louis, MO, USA), dissolved in Hanks’ balanced solution at a concentration of 5 mg/ml, was added to each well with the indicated treatment and incubated for 4 h. Finally, the medium was aspirated from each well, and 150 μl of dimethyl sulfoxide (Veterco, Thoi Binh Ward, Can Tho, Vietnam) was added to dissolve formazan crystals, and the absorbance of each well was obtained using a Dynatech MR5000 plate reader at 490 nm with a reference wavelength of 630 nm. If the cells were cultivated for 72h, we set the cell viability (viable cells) of the control group at 72h as to 100% and the percentage of cell viability (viable cells) in other groups to the control group at 72h were calculated. If the cells were cultivated for 48h, we set the cell viability (viable cells) of the control group at 48h as to 100% and the percentage of cell viability (viable cells) in other groups to the control group at 48h were calculated.

### 2.4 Western blot

After different treatments, the experimental cells were lysed in ice-cold lysis buffer [50 mM Tris–HCl pH 8.0, 150 mM NaCl, 1% NP40, 0.05% SDS, 10 mM NaF, 1 mM PMSF, 2 mM Na_3_VO_4_, and complete protease inhibitor cocktail tablet (Roche)], and cleared by microcentrifugation. Aliquots of 50 μg of protein from each sample were separated by 10–12% SDS-PAGE and transferred onto a PVDF membrane (Millipore, Bedford, MA). After blocking with 10% instant nonfat dry milk for 1 h, membranes were incubated with specific antibodies overnight at 4°C, followed by incubation with secondary antibodies for 2 h. The blots were developed using the ECL system (Immobilion™ Western, Millipore) and were analyzed by Quantity One Software (Bio-Rad, USA). Protein contents were normalized by β-Actin (Santa Cruz) level and the final data were a relative density to that in control/controlsiRNA group.

### 2.5 cAMP content and PKA activity assay

Quantification of cAMP and PKA activity were carried out using the Cyclic AMP ELISA Kit (ab138880) and PKA Kinase Activity Assay Kit (ab139435) (Abcam, Shanghai, China) following manufacturer’s instructions and previous report [10, 11].

### 2.6 RNA interference (RNAi)

SiRNA for AMPKα1 and LKB1 were purchased from Santa Cruz Biotechnology, Inc. (CA. USA). SiRNA for CNR2 and CaMKKβ, Lipofectamine RNAiMAX reagent and opti-MEM were purchased from Invitrogen (Shanghai, China). 6 pmol RNAi duplex was diluted in 100 ul opti-MEM without serum in the wells of the tissue culture plate. 1 ul Lipofectamine RNAiMAX was added to each well containing the diluted RNAi molecules, followed by gent mixing and incubation for 10-20 minutes at room temperature. C6 cells were diluted in complete growth medium without antibiotics at 3×10^4^ cells/500 ul, to make the final volume 600 ul with a final RNA concentration of 10 nM. Target protein expression was determined by western blot at 48 h after transfection, and successfully knocked down cells were used for further experiments.

## 3 Results

### 3.1 JWH133 inhibited C6 cells growth in a dose-dependent manner

**Figure 1:**
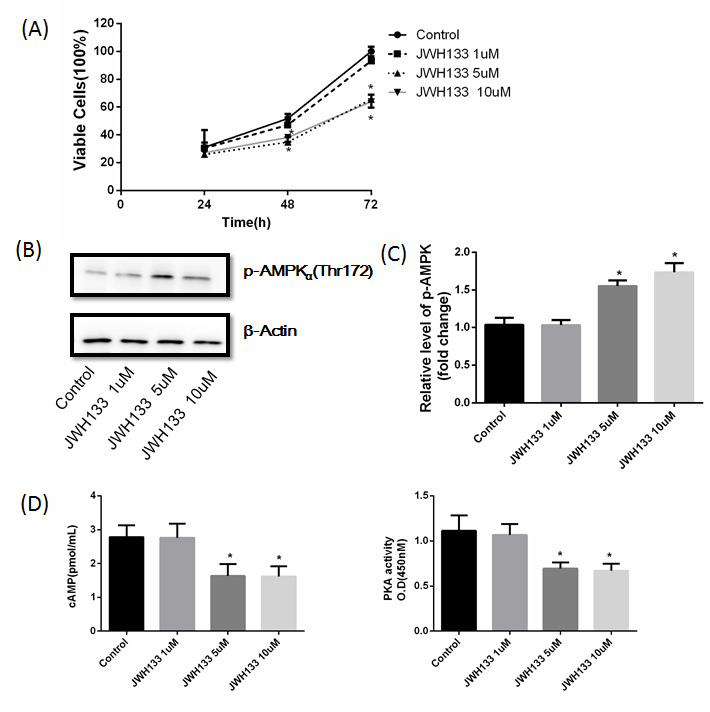
**JWH133 inhibited C6 cells growth in a dose-dependent manner**. (A) C6 cells were treated with different doses of JWH133 (1, 5 and 10 μM) at different time points (24, 48 and 72 h). Cell viability was analyzed by 3-[4,5-dimethylthiazolyl-2] 2,5-diphenyl-tetrazolium bromide (MTT) test. The original western blot images (B) and statistic graph (C) of p-AMPKα affected by JWH133 (1, 5 and 10 μM) for 48h. Data were relative in density to that in control group. (D) cAMP content and PKA activity were reduced in a dose-dependent manner, when C6 cells were administrated of JWH133 (1, 5 and 10 μM) for 48 h. Data were mean ± SEM from three replicate experiments, *P<0.05 versus control (at the same time).

C6 cells were treated with 1, 5 and 10 μM JWH133 for 24, 48 and 72 h respectively. JWH133 (1 μM) had no effect on C6 cells growth, p-AMPKα (Thr172), cAMP content and PKA activity. While JWH133 5μM and 10 μM both inhibited C6 cells growth at 48h and 72 h. JWH133 5μM and 10 μM both increased p-AMPKα (Thr172), and decreased cAMP content and PKA activity at 48h (Fig. 1). We therefore chose 5 μM as the optimal dose of JWH133 in the following experiments.

**Figure 2:**
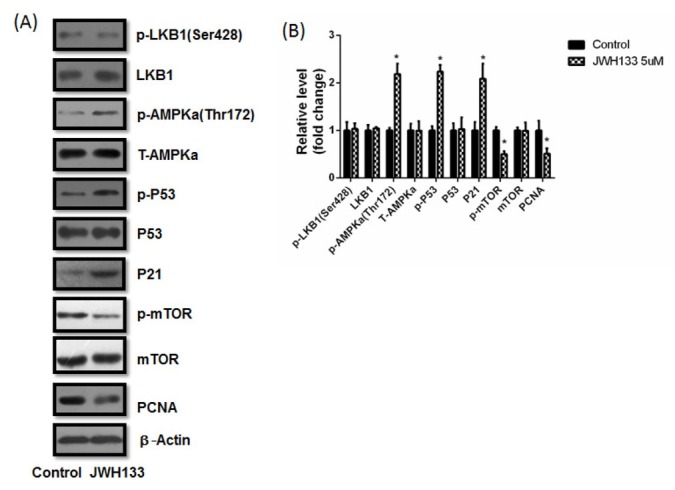
**Relative proteins variation in C6 cells administration of JWH133 (5** μ**M) for 48 h**. JWH133 elevated the expressions of p-AMPKα (Thr172), p-P53 and P21, reduced the expressions of p-mTOR and PCNA, while had no effect on the expressions of p-LKB1 (Ser428), LKB1, T-AMPKα, P53, mTOR and β-Actin. The original western blot images (A) were representative of three different experiments. (B) The statistic graphs of proteins variation. Data were relative density to that in control group. Data were mean ± SEM from three replicate experiments, *P<0.05 versus control. The samples were derived from the same experiment and that blots were processed in parallel. The grouping of blots in the same protein came from the same gel and were not cropped, but grouping blots of different protein came from different gels. All the following blots adhere to this rule.

Next, C6 cells were treated with 5 μM JWH133 for 48 h. The expression of p-AMPKα (Thr172), p-P53 and P21 increased, while p-mTOR and PCNA decreased. On the other hand, p-LKB1, total LKB1, total AMPKα (T-AMPKα), total P53 and total mTOR did not change at all (Fig. 2).

### 3.2 The effects of JWH133 on C6 cells were mediated through CB2 receptor

**Figure 3:**
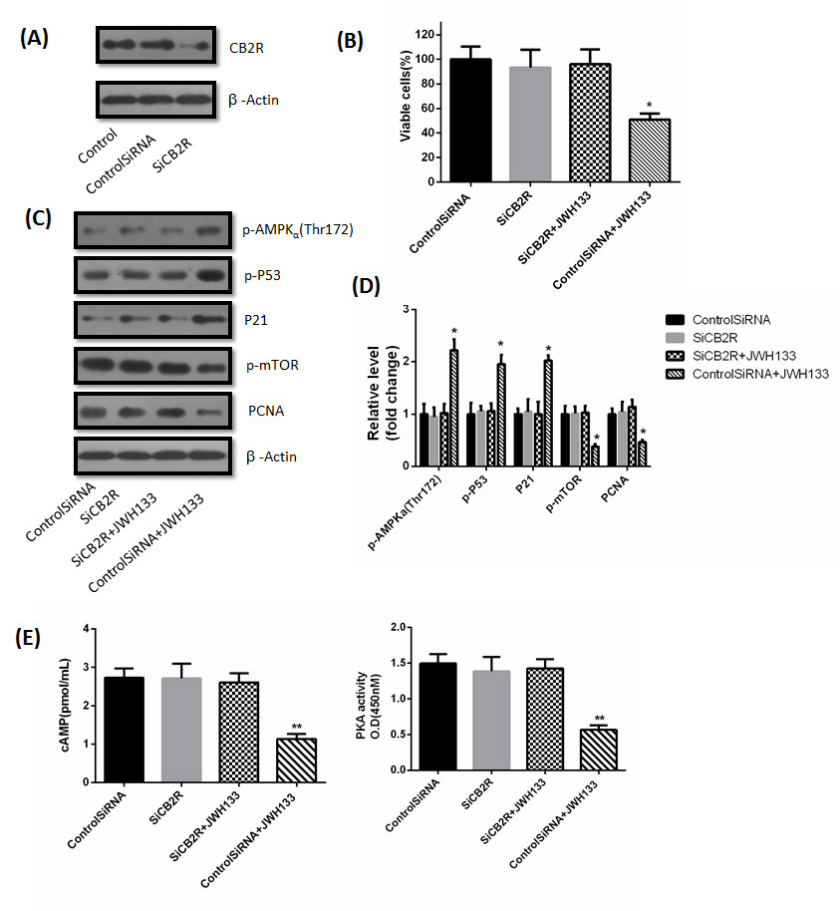
**Knocking down cannabinoid receptor 2 (CB2) receptors by small interference RNA inhibited the effects of JWH133 on C6 cells**. (A) C6 cells were transfected with small interfering control RNA (Control SiRNA) or CB2 receptors RNA (SiCB2R) for 24 h. CB2 receptor and β-Actin expression levels were detected by Western blot. CB2 receptor knocked down cells were used for further experiments. The image was representative of three different experiments. (B) Effects of JWH133 (5 μM) on the viability (48 h) of C6 cells transfected with Control SiRNA or SiCB2R. The original western blot images (C) and statistic graphs (D) of p-AMPKα (Thr172), p-P53, P21, p-mTOR and PCNA expression in Control SiRNA or SiCB2R-transfected C6 cells administration of JWH133 (5 μM, 48 h). Data were relative density to that in Control SiRNA group. (E) The effects of JWH133 on cAMP content and PKA activity were abolished by transfecting SiCB2R to C6 cells. Data were mean ± SEM from three replicate experiments. *P<0.05, **P<0.01 versus Control SiRNA.

C6 cells were transfected with small interfering CB2 receptor RNA (SiCB2R) and control siRNA for 24 h. SiCB2R efficiently downregulated CB2 receptor in C6 cells, whereas control siRNA had almost no effect on CB2 receptor expression (Fig. 3A). While CB2 receptor silencing by SiCB2R had no effect on cell viability (48h), it obviously abrogated the JWH133 (5 uM)-induced inhibition of C6 cells viability (48h) (Fig. 3B). Similarly, silencing CB2 receptor had no effects on expression of p-AMPKα (Thr172), p-P53, P21, p-mTOR and PCNA (48h), but abrogated the inhibitory effects of JWH133 (5uM) on PCNA and p-mTOR (48h), as well as the promotional effects of JWH133 (5uM) on p-AMPKα (Thr172), p-P53 and P21 (48h) (Fig. 3 C and D). Similarly, silencing CB2 receptor had no effects on cAMP content and PKA activity (48h), but abolished the inhibitory effects of JWH133 (5uM) on cAMP content and PKA activity (48h) (Fig. 3E).

**Figure 4:**
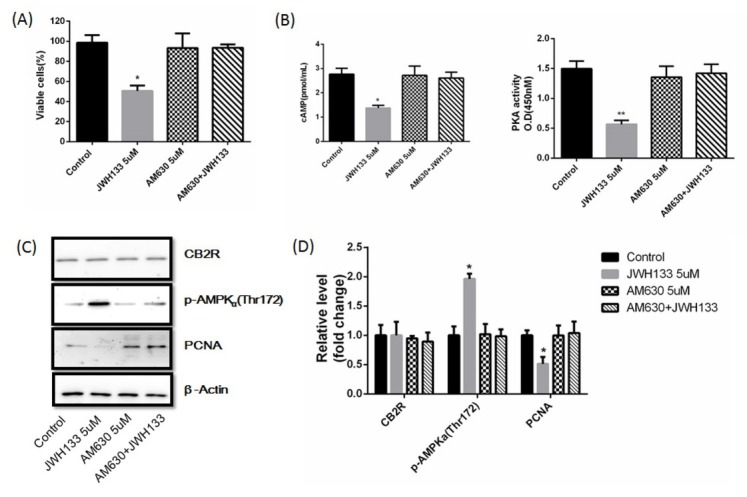
**The effects of JWH133 (5 uM, 48h) on C6 cells were abolished by CB2 receptor antagonist AM630 (5 uM)**. (A) The effects of JWH133 (5 μM) on the viability (48 h) of C6 cells were abolished by AM630 (5 uM). (B) The effects of JWH133 on cAMP content and PKA activity were abolished by AM630. The original western blot images (C) and statistic graphs (D) of CB2 receptor, p-AMPKα (Thr172) and PCNA expression in JWH133 and AM630 treated C6 cells. Data were relative density to that in Control group. Data were mean ± SEM from three replicate experiments. *P<0.05, **P<0.01 versus Control.

The CB2 receptor antagonist AM630 had no effects on C6 cells viability, cAMP content, PKA activity, CB2 receptor expression, p-AMPKα (Thr172) and PCNA. But AM630 abolished the inhibitory effects of JWH133 (5 uM) on C6 cells viability (48h), abolished the inhibitory effects of JWH133 (5uM) on cAMP content and PKA activity (48h), abolished the promotional effects of JWH133 (5uM) on p-AMPKα (Thr172) (48h), and abolished the inhibitory effects of JWH133 (5uM) on PCNA (48h). (Fig. 4)

**Figure 5:**
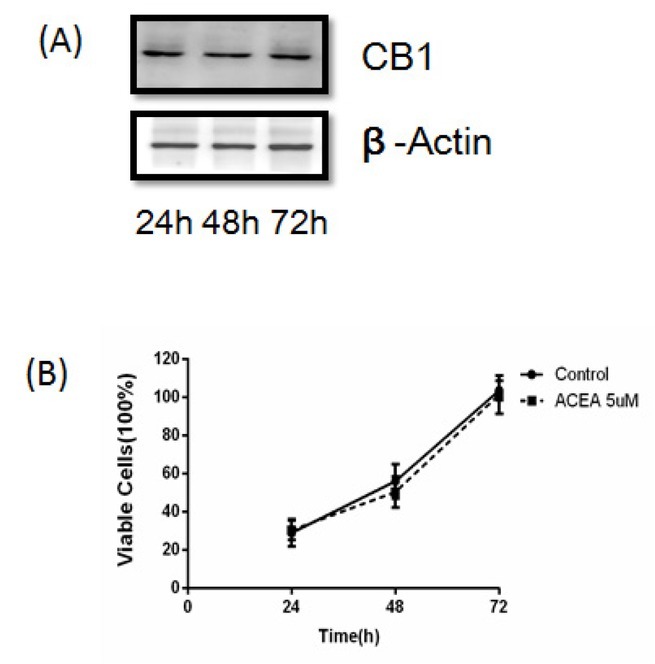
**CB1 receptor agonist ACEA (5uM) had no effects on C6 cells proliferation**. (A)CB1 receptor and β-Actin expression were detected by Western blot. There were no changes on the CB1 receptor expression in C6 cells cultivated for 24h, 48h and 72h. (B) ACEA (5 uM) had no effects no viability of C6 cells. Data were mean ± SEM from three replicate experiments. *P<0.05, **P<0.01 versus Control.

CB1 receptors also expressed on the C6 cells (Fig. 5A), CB1 receptor agonist ACEA 5uM had no effect on cell viability at 24h, 48h and 72h (Fig. 5B). That proved CB1 receptor had nothing to do with the C6 cells viability, so effects of JWH133 on C6 cells were not mediated through CB1 receptor.

### 3.3 AMPK activation was involved in the effects of JWH133 on C6 cells, except for the effects on cAMP content and PKA activity

**Figure 6:**
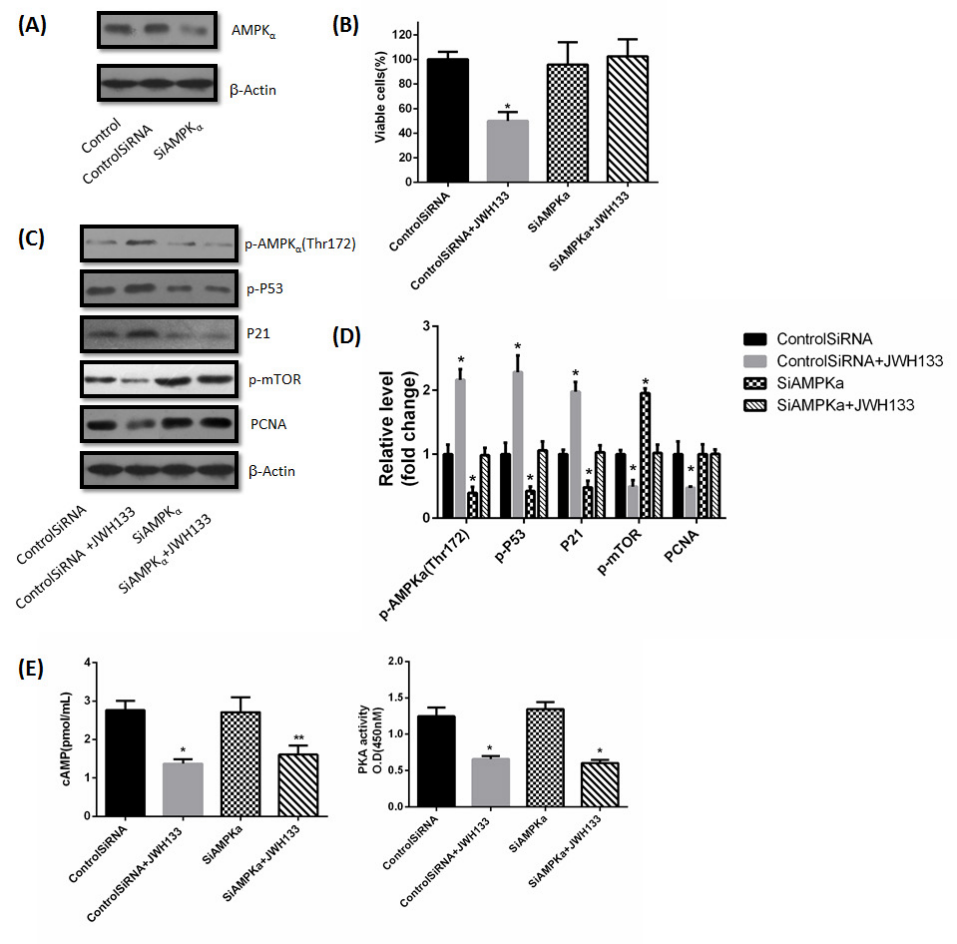
**The effects of JWH133 on C6 cells had nothing to do with LKB1**. (A) C6 cells were transfected with small interfering control RNA (Control SiRNA) or LKB1 RNA (SiLKB1) for 24 h. LKB1 and β-Actin expression levels were detected by Western blot. LKB1 knocked down cells were used for further experiments. The image was representative of three different experiments. (B) Effects of JWH133 (5 μM) on the viability (48 h) of C6 cells transfected with Control SiRNA or SiLKB1. The original western blot images (C) and statistic graphs (D) of LKB1, p-LKB1, p-AMPKα (Thr172) and PCNA expression in Control SiRNA or SiLKB1-transfected C6 cells administration of JWH133 (5 μM, 48 h). Data were relative density to that in Control SiRNA group. Data were mean ± SEM from three replicate experiments. *P<0.05, **P<0.01 versus Control SiRNA.

C6 cells were transfected with small interfering AMPKα RNA (SiAMPKα) and control siRNA for 24 h. SiAMPKα efficiently knocked down AMPKα in C6 cells, whereas control siRNA had almost no effect on AMPKα expression (Fig. 6A). Silencing AMPKα by SiAMPKα had no effect on C6 cells viability itself, but obviously abolished the JWH133 (5uM)-induced inhibition of C6 cells viability (48h) (Fig. 6B). Similarly, silencing AMPKα had no effect on PCNA, but abrogated the inhibitory effect of JWH133 (5uM) on PCNA (48h). AMPKα silencing effectively reduced p-AMPKα (Thr172), p-P53 and P21, while increased p-mTOR (48h) (Fig. 6 C and D). These observations supported that P53, P21 and mTOR are the downstream targets of AMPK activation, the results are consistent with previous reports. Silencing AMPKα efficiently abrogated JWH133 (5uM) -induced p53 activation (phosphorylation at Ser-15 and accumulation) and p21 induction (48h), and abolished the inhibitory effect of JWH133 (5uM) on p-mTOR (48h) (Fig. 6 C and D). Silencing AMPKα had no effects on cAMP content and PKA activity (48h), and did not abolish the inhibitory effects of JWH133 (5uM) on cAMP content and PKA activity (48h) (Fig. 6E). These data indicated that cAMP-PKA is the upstream activator of AMPKα.

### 3.4 Activation of AMPK by JWH133 required CaMKKβ, but not LKB1

**Figure 7:**
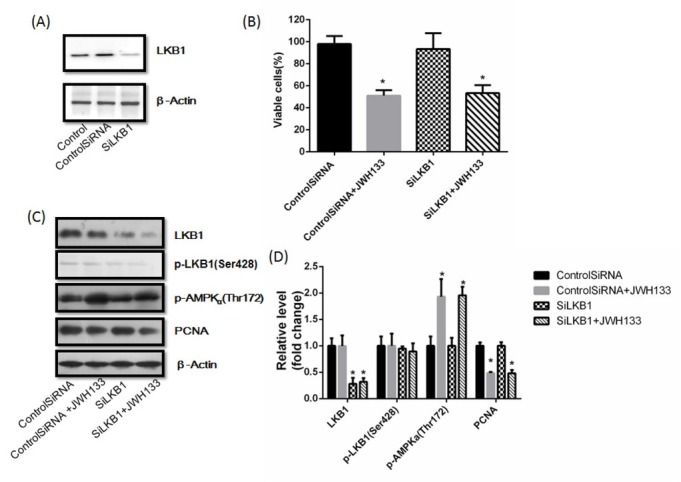
**JWH133 activated AMPK and related downstream proteins to inhibit C6 cells proliferation**. (A) C6 cells were transfected with small interfering control RNA (Control SiRNA)or AMPKα RNA (SiAMPKα) for 24 h. AMPKα and β-Actin expression levels were detected by western blot. AMPKα knocked down cells were used for further experiments. The image was representative of three different experiments. (B) Effect of JWH133 (5 μM) on the viability (48 h) of C6 cells transfected with Control SiRNA or SiAMPKα. The original western blot images (C) and statistic graphs (D) of p-AMPK (Thr172), p-P53, P21, p-mTOR and PCNA expression in Control SiRNA or SiAMPKα-transfected C6 cells administration of JWH133 (5 μM, 48 h). Data were relative density to that in Control SiRNA group. (E) The effects of JWH133 on cAMP content and PKA activity had nothing to do with AMPKα. Data were mean ± SEM from three replicate experiments, *P<0.05, **P<0.01 versus control SiRNA.

We next investigated the mechanism by which JWH133 activated AMPK. Among different kinases proposed to act as AMPKKs, the human tumor suppressor liver kinase B1 (LKB1) and the calmodulin-activated kinase kinase (CaMKK) are widely accepted as the most relevant ones. In our results, JWH133 did not affect LKB1 and p-LKB1 expression in C6 cells (Fig. 7C and D), and selective knockdown of LKB1 by LKB1 siRNA did not abolish the inhibition of JWH133 on C6 cells viability (Fig. 7B). Silencing LKB1 had no effects on expression of PCNA and p-AMPKα (Thr172), and also did not abolish JWH133-induced effect on PCNA and p-AMPKα (Thr172) (Fig. 7C and D). This suggested that LKB1 was not involved in JWH133-induced AMPK activation.

**Figure 8:**
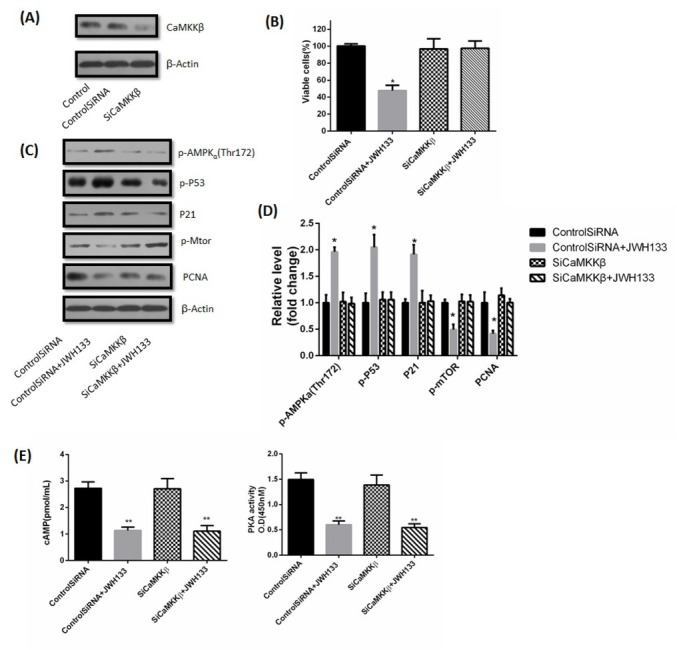
**JWH133 activated AMPK via CaMKK**β. (A) C6 cells were transfected with small interfering control RNA (Control SiRNA) or CaMKKβ RNA (SiCaMKKβ) for 24 h. CaMKKβ and β-Actin expression levels were detected by Western blot. CaMKKβ knocked down cells were used for further experiments. The image was representative of three different experiments. (B) Effect of JWH133 (5 μM) on the viability (48 h) of C6 cells transfected with Control SiRNA or SiCaMKKβ. The original western blot images (C) and statistic graphs (D) of p-AMPK (Thr172), p-P53, P21, p-mTOR and PCNA expression in Control SiRNA or SiCaMKKβ-transfected C6 cells administration of JWH133 (5 μM, 48 h). Data were relative density to that in Control SiRNA group. (E) The effects of JWH133 on cAMP content and PKA activity had nothing to do with CaMKKβ. Data were mean ± SEM from three replicate experiments, *P<0.05, **P<0.01 versus Control SiRNA.

By contrast, selective knockdown of CaMKKβ by CaMKKβ siRNA prevented the inhibition of JWH133 on C6 cells viability (Fig. 8B). Similarly, silencing CaMKKβ had no effects on expression of PCNA, p-AMPKα (Thr172), p-P53, P21 or p-mTOR, but abrogated JWH133-induced inhibitory effect on PCNA and mTOR, as well as promotional effects of JWH133 on p-AMPKα, P21 and p-P53 (Fig. 8C and D).

These indicated that AMPK activation by JWH133 in C6 cells relied on CaMKKβ. The effects of JWH133 on cAMP content and PKA activity were not abolished in SiCaMKKβ-transfected C6 cells (Fig. 8E). This indicated that cAMP-PKA was also the upstream activator of CaMKKβ.

### 3.5 Activation of CaMKKβ was related to inhibition of cAMP-PKA activity

**Figure 9:**
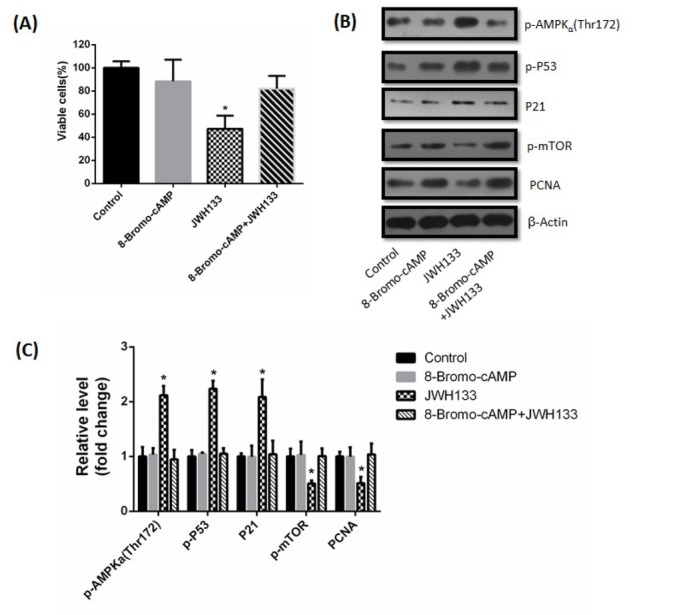
**8-Bromo-cAMP abolished the effects of JWH133 on C6 cells**. (A) Effect of JWH133 (5 μM) on the viability (48 h) of C6 cells incubated in the presence or absence of the 1 μM 8-Bromo-cAMP. The original western blot images (B) and statistic graphs (C) of p-AMPK (Thr172), p-P53, P21, p-mTOR and PCNA expression in C6 cells administration of JWH133 (5 μM, 48 h) with or without 8-Bromo-cAMP (1 μM). Data were relative density to that in Control group. The image was representative of three different experiments. Data were the mean ± SEM of three different experiments, *P<0.05 versus control.

**Figure 10:**
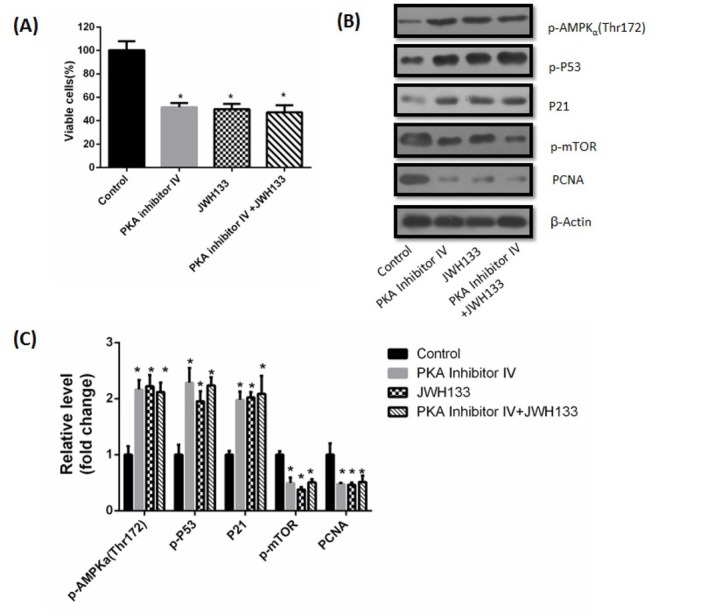
**PKA inhibitor IV did not abolish the effects of JWH133 on C6 cells**. (A) Effect of JWH133 (5 μM) on the viability (48 h) of C6 cells incubated in the presence or absence of the 500 nM PKA Inhibitor IV. The original western blot images (B) and statistic graphs (C) of p-AMPK (Thr172), p-P53, P21, p-mTOR and PCNA expression levels in C6 cells administration of JWH133 (5 μM, 48 h) with or without PKA inhibitor IV (500 nM). Data were relative density to that in Control group. Data were the mean ± SEM of three different experiments, *P<0.05 versus control.

The above results showed that cAMP-PKA was the upstream activator of CaMKKβ and AMPK. Next we investigated the effects of cAMP-PKA on CAMKKβ and AMPKα activation, we used the cAMP analog “8-Bromo-cAMP” and the PKA inhibitor IV to activate and inhibit PKA, respectively. C6 cells were incubated with 1 μM 8-Bromo-cAMP for 48 h, promotional effects of JWH133 on p-AMPKα, P21 and p-P53 and inhibitory effects on p-mTOR were all abrogated (Fig.9 B and C). At the same time, incubation of 8-Bromo-cAMP also prevented the JWH133-induced inhibition of C6 cells viability and PCNA expression (Fig. 9). These results showed that 8-Bromo-cAMP activated PKA, thereby abrogated the effects of JWH133 on C6 cells. In contrast, incubation with PKA inhibitor IV (500 nM) did not affect the JWH133-induced effects on C6 cells. But PKA inhibitor IV itself mimicked the effects of JWH133 on C6 cells, as evidenced by a decrease of cell viability and PCNA expression, increase of p-AMPKα, P21 and p-P53, as well as inhibition of p-mTOR (Fig. 10).

## 4 Discussion

In this study, we have shown that the CB2 receptor-selective agonist JWH133 inhibited C6 cells growth. We also confirmed that JWH133 activated CB2 receptor to reduce cAMP production and inhibit PKA activity. Decreased PKA activity stimulated CaMKKβ, then elevated AMPKα phosphorylation at threonine 172 site. The activation of AMPK then induced changes in downstream proteins, including increased P53 phosphorylation and P21 expression, as well as decreased mTOR phosphorylation, which eventually inhibited C6 cells growth.

Studies have shown that CB2 receptors are present in both cultured neurons and the nervous system [12, 13]. Co-expression of the CB1 and CB2 cannabinoid receptors has been detected in rat C6 glioma cells and in biopsies from human astrocytomas [4]. Moreover, the extent of CB2 expression is related to tumor progression. Another study indicated that a high level of CB2 receptor subtype in biopsies of human astrocytomas and glioblastomas, also appears to be correlated with tumor malignancy [5]. Calatazzolo et al. found higher expression of CB2 receptors in glioblastomas and endothelial cells than in low-grade gliomas [14]. High levels of CB2 expression in either gliomas or endothelial cells of glioblastoma vessels are also demonstrated by Schley et al. [15], which suggests that these tumors can be vulnerable to a cannabinoid treatment thereby hinting at a CB2 cannabinoid specific agonist-based strategy. JWH-133 is a selective agonist for the CB2 receptor, and has been investigated as a possible cancer therapeutic agent with low psychoactive side effects. The first study to demonstrate the cytotoxicity of JWH-133 in vitro is conducted in glioma cells [4], where JWH-133 reduces cell viability by 50% in vitro. In addition, in vivo studies showed a 71% decrease in tumor growth after 8 days in Rag_−/−_ mice with 40 μg/day intratumor administration of JWH133. This effect on tumor growth is inhibited by the CB2 antagonist SR144528, but not the CB_1_-specific antagonist SR141716 [4]. These results are consistent with studies in C6 glioma cells and astrocytoma xenografts, where the overall vascularization of tumors is reduced by 88% and 21%, respectively [16]. Our study also found that JWH133 inhibited C6 cell growth, which could be abolished by silencing CB2 receptor or AM630. Furthermore in our experiment, CB1 receptor agonist had no effect on cell viability, so effects of JWH133 on C6 cells were not mediated through CB1 receptor. Therefore, JWH133 inhibits C6 cells growth via CB2 receptors, which is consistent with the above previous reports.

We also found that JWH133 activated AMPK and increased the phosphorylation of AMPKα at Thr172 site. Vara et al. reported that anti-proliferative action of CB2 agonist JWH-015 in HCC cells depends on stimulating AMPK [8]. Choi et al. reported that CB2 receptor activation by trans-caryophyllene ameliorates ischemic injury, potentially through the AMPK/CREB pathway [9]. Kamikubo R et al. reported that β-caryophyllene-induced activation of AMPK is mediated by CB2 receptor [17]. In line with these observations, we found that CB2 receptor silencing abrogated the promotional effect of JWH133 on p-AMPKα (Thr172). Therefore, we speculate that JWH133 activates AMPK via CB2 receptors.

Our study also showed that the activation of AMPK by JWH133 increased P53 phosphorylation and P21 expression, decreased mTOR phosphorylation and reduced cell viability, while silencing AMPKα, effectively abrogated these JWH133-induced effects. Many studies have demonstrated that AMPK activation is involved in p53 activation (phosphorylation at Ser-15/upregulation) and p21 upregulation. For example, Zhang et al. reported AMPK activation is involved in TMZ-induced p-p53 and p21 upregulation7. Activation of AMPK by AICAR suppresses the growth of HepG2 hepatoma cells and induces expression of wild-type p53 and p21 [18]. AMPK activation also enhances p53 phosphorylation/activation, which subsequently induces apoptosis [19, 20]. In addition, ectopic expression of wild-type AMPK kinase LKB1 increases p21 concentration and inhibits the growth of LKB1-deficient A549 lung adenocarcinoma cells [21, 22]. Apart from positively regulating p53 and P21, AMPK activation might also induce tumor cell death or inhibit tumor cell growth by inhibiting mTOR signaling [7, 23]. In our study, silencing AMPKα efficiently reduced p-AMPKα (Thr172), P53 phosphorylation and P21 expression, but increased mTOR phosphorylation. These observations also suggest that P53, P21 and mTOR are the downstream targets of AMPK activation, consistent with previous reports. We therefore speculate that JWH133 activates AMPK via CB2 receptors. Activation of AMPK then induces changes in downstream proteins, such as increased P53 phosphorylation and P21 expression, decreased mTOR phosphorylation, which consequently inhibit C6 cells growth. Further investigations are needed to reveal the relationship between P53, P21 and mTOR.

How does activation of CB2 receptor by JWH133 lead to the activation of AMPK? We investigated the mechanism between CB2 receptor and AMPK. The gene encoding the human cannabinoid CB2 receptor was cloned in 1993 [24]. It belongs to the seven-transmembrane-domain, G-protein-coupled receptor class [25], and can modulate various signal transduction pathways controlling cell proliferation, differentiation and survival. Thus, by coupling to Gi/o proteins, the CB2 receptor inhibits adenylyl cyclase and the cAMP pathway in various types of cells expressing the receptors either naturally or heterologously [25]. LKB1 is regulated by intermediates of GPCR signaling, specifically PKA, which phosphorylates LKB1 [26, 27]. It has been speculated that elevations in cyclic AMP can result in increased LKB1 phosphorylation by PKA, which enhances LKB1-AMPK interactions. Therefore, cyclic AMP, by activating PKA, can lead to activation of LKB1 and consequent phosphorylation of AMPK at Thr172 [28]. In our study, we found that activating CB2 receptor by JWH133 indeed decreased cAMP content and PKA activity, but had no effects on LKB1 phosphorylation, suggesting LKB1 is not involved in the activation of AMPK by JWH133. In addition, CAMKK can be phosphorylated and inhibited by PKA [29], likely allowing Gs- and Gi-coupled receptors to regulate AMPK activity through a CAMKK-dependent pathway. Our study showed that CaMKKβ silencing abrogated JWH133-induced activation of AMPK, indicating that AMPK activation by JWH133 in C6 cells relies on CaMKKβ.

The adenylate cyclase activator forskolin has been reported to inhibit AMPK activity. The mechanism involves activating PKA to inhibit CaMKK, which in turn may limit AMPK activation in response to energy depletion [30]. Racioppi also reported that cAMP/PKA pathway can inhibit CaMKKβ activity [31]. Our study showed that JWH133 inhibited cAMP-PKA activity through CB2 receptor, then activated CaMKKβ, suggesting cAMP-PKA is the upstream signal of CaMKKβ. We also found that 8-Bromo-cAMP, but not PKA inhibitor IV, abrogated JWH133-induced activation of AMPK. It is interesting that PKA inhibitor IV itself mimicked the effects of JWH133 on C6 cells, by increasing AMPK phosphorylation. We therefore postulate that the reduction of PKA by JWH133 relieves CaMKK inhibition, suggesting JWH133 activates CaMKK by reducing cAMP-PKA activity.
